# Sociodemographic and Anthropometric Factors Associated With Screen-Based Sedentary Behavior Among Japanese Adults: A Population-Based Cross-Sectional Study

**DOI:** 10.2188/jea.JE20130008

**Published:** 2013-09-05

**Authors:** Kaori Ishii, Ai Shibata, Koichiro Oka

**Affiliations:** Faculty of Sport Sciences, Waseda University, Tokorozawa, Saitama, Japan; 早稲田大学スポーツ科学学術院

**Keywords:** weight status, Japanese, sedentary behavior, sociodemographic

## Abstract

**Background:**

Concern over the health risks of sedentary behavior has highlighted the need to examine factors associated with screen-based (television/computer) sedentary behavior. The present study examined the association of screen-based sedentary behavior with body weight and sociodemographic attributes among Japanese adults.

**Methods:**

A population-based cross-sectional study enrolled 1034 Japanese adults aged 40 to 69 years who lived in 2 Japanese cities. Sociodemographic variables, height, weight, and time spent on screen-based sedentary behavior were collected by self-administered questionnaire. Differences in screen time in relation to body mass index and weight gain since age 20 years were assessed by the Mann-Whitney U test. Independent associations of each variable with screen time were examined by forced-entry logistic regression analyses.

**Results:**

Mean (SD) age and median (interquartile range) duration of screen time per week were 55.6 (8.4) years and 832.0 (368.8–1263.1) minutes, respectively, for men, and 55.3 (8.4) years and 852.6 (426.0–1307.5) minutes, respectively, for women. Screen time among participants with weight gain was longer than among those with a weight gain of less than 10 kg (*P* = 0.08). Unmarried and unemployed participants had longer screen times. Participants aged 40 to 49 years were less likely than older age groups to spend time on screen-based sedentary behavior during leisure hours.

**Conclusions:**

The present findings imply that strategies are necessary to discourage screen-based sedentary behavior among all demographic groups, especially among adults who are elderly, unmarried, or unemployed.

## Abstract (ja)

**背景:**

健康リスクに影響を与えるスクリーンタイム(テレビやコンピューターの使用)の座位行動に関連する要因の検討が求められている。そこで本研究では、日本人成人におけるスクリーンタイムの座位行動と体重・体格および社会人口統計学的要因との関連を検討した。

**方法:**

日本の2つの地域に居住する40～69歳の地域住民1,034名に対し横断調査を実施した。自己記入式質問紙にて社会人口統計学的要因、身長、体重およびスクリーンタイムの座位行動時間を調査した。BMIおよび20歳時からの体重増加によるスクリーンタイムの違いをマン・ホイットニーのU検定にて検討した。また、ロジスティック回帰分析により、スクリーンタイムに関連する社会人口統計学的要因を検討した。

**結果:**

平均年齢(標準偏差)は男性で55.6(8.4)歳、女性で55.3(8.4)歳、週あたりのスクリーンタイムの中央値(25パーセントタイル–75パーセントタイル)は男性で832.0(368.8–1263.1)分、女性で852.6(426.0–1307.5)分であった。20歳からの体重増加が10kg以上の者は、10kg未満の者よりスクリーンタイムが長かった(*P* = 0.08)。独身者、仕事に就いていない者は、よりスクリーンタイムが長かった。また、40～49歳の者はそれ以上の者よりもスクリーンタイムが短かった。

**結論:**

本研究の結果より、日本人成人におけるスクリーンタイムの座位行動に関連する体重・体格および社会人口統計学的要因が特定された。本研究の結果は、特に高齢者、独身者、仕事に就いていない者に対するスクリーンタイムの座位行動を減少させるための対策の必要性を示している。

## INTRODUCTION

Recent studies have reported that the amount of leisure time spent on discretionary screen-based sedentary behavior (as opposed to that required during working hours), such as television viewing and computer use, is associated with increased risks of weight gain, type II diabetes, and cardiovascular disease.^1^^–^^10^ Sedentary behavior is defined as activity that involves energy expenditure in the range of 1.0 to 1.5 metabolic equivalents (METs).^11^ The mean time spent by adults on screen-based sedentary behavior was reported to be 188.0 minutes/day in Scotland^12^ and 235.1 minutes/day in Australia.^13^ Although screen-based sedentary behavior is associated with health risks, a large proportion of the population in developed countries is sedentary. Reducing sedentary time increases opportunities to be physically active, so it is important to focus on screen-based sedentary behavior.

Physical activity guidelines^14^^,^^15^ and a recent global statement^16^ encourage a widespread, population-based approach to decrease sedentary behavior. The guidelines emphasize the consequences of sedentary behavior and the need to tailor interventions to the requirements of specific population groups such as children, adults, men, women, older persons, disabled persons, employees, and diverse cultural groups.^14^^–^^16^ Determining the amount of leisure time spent by specific population groups on screen-based sedentary behavior is also important when designing strategies to decrease such behavior.

Screen-based sedentary behavior has been investigated among various demographic groups, especially in the United States, Australia, and Europe. A systematic review found that relationships between sedentary behavior and sociodemographic status vary by country.^17^ Many studies on the correlates of sedentary behavior have been conducted among children, but studies of adults are less common. The sociodemographic factors associated with screen-based sedentary behavior among Japanese adults remain to be determined. In addition, associations of anthropometric factors such as body mass index (BMI) and weight gain with screen-based sedentary behavior were less evident in the Japanese population. The present study examined the associations of sociodemographic and anthropometric factors with screen-based sedentary behavior among adults in Japan.

## METHODS

### Participants and data collection

The present cross-sectional study was conducted from February through March 2011. A total of 3000 residents aged 40 to 69 years and living in 2 Japanese cities, Kanuma and Nerima, were randomly selected from the residential registries of those cities. Potential participants were stratified by sex and age bracket (40–49, 50–59, and 60–69 years). The plan was to include 1500 participants of each sex, 1000 participants from each age bracket, and 1500 participants from each city. Nerima is an urban area within commuting distance of Tokyo, while Kanuma is a mid-sized city in central Japan. These 2 cities were chosen to reflect urban and suburban lifestyles.

A self-administered questionnaire that included questions on sociodemographic variables, sedentary behavior, height, and weight was mailed to participants. To encourage a response, letters explaining the study were sent to all participants 2 weeks before the questionnaire. Nonresponders were sent 1 reminder about the questionnaire. A total of 1105 participants replied to the questionnaire (36.8% overall response rate; 33.8% from Kanuma and 37.9% from Nerima). All participants signed an informed consent document before answering the questionnaire. The Ethics Committee of Waseda University, Japan approved the study before its commencement.

### Measures

#### Sociodemographic factors

Participants provided information on sociodemographic factors such as sex, age, education level, employment status, marital status, living arrangements, and household income level by choosing the most suitable response from a set of predetermined options, as follows: education level (graduate school or university, 2 years of university education or equivalent, college, high school, or junior high school), employment status (full-time, part-time, full-time homemaker, student, or unemployed), marital status (married or unmarried), living arrangements (cohabitating or living alone), and household income level (<3, ≥3–<5, ≥5–<7, ≥7–<10, or ≥10 million yen).

#### Anthropometric variables

BMI was calculated from self-reported height and weight. Weight gain since age 20 years was estimated from self-reported weight at age 20 years.

#### Screen-based sedentary behavior

Participants reported duration of screen-based sedentary behavior over a usual week (screen time). Screen-based sedentary behavior comprised computer and Internet use for leisure purposes, television watching, computer gaming, and video or DVD watching.^13^^,^^18^ Participants were asked how many times per week they spent on screen time and the duration of screen time on each of those days. The scale was previously shown to have acceptable reliability and validity.^18^ The test–retest reliability of the items was found to be moderate (range 0.6–0.8). Validity, as defined by correlations with 3-day behavioral log data, was moderate (range 0.3–0.6). The total time spent on screen time was classified as low or high, based on the median of 841.8 minutes/week.

### Statistical analyses

Differences in participant characteristics and screen time were compared by sex and city with the *t*-test, χ^2^ test, and Mann-Whitney U test. Differences in screen time between those with a BMI of less than 25 kg/m^2^ versus 25 kg/m^2^ or higher, and those with a weight gain of less than 10 kg versus 10 kg or more, since age 20 were assessed by the Mann-Whitney U test. Forced-entry logistic regression analyses were conducted to examine independent relationships of each sociodemographic variable with screen time. Responses to sociodemographic attributes were categorized as follows: sex (male or female), age (40–49, 50–59, and 60–69 years), education level (≥4 years of university education, 2 years of university education or equivalent, or high school or junior high school), employment status (employed or not employed), marital status (married or unmarried), living arrangements (living with another individual or living alone), and household income level (<3, ≥3–<5, ≥5–<7, ≥7–<10, or ≥10 million yen). All statistical analyses were performed with SPSS 18.0J for Windows (Statistical Package for the Social Sciences; SPSS Inc. Chicago, IL, USA). A *P* value of less than 0.05 was considered to indicate statistical significance.

## RESULTS

### Sociodemographic characteristics of participants

Data from the 1034 adults (540 men and 494 women) who fully completed the survey were included in the analysis. Table 1 shows the sociodemographic characteristics of the study participants. Mean (SD) age for men was 55.6 (8.4) years, and mean age for women was 55.3 (8.4) years. The proportions of married male and female respondents were 84.2% and 85.0%, respectively; 91.3% and 94.1%, respectively, lived with other people and 85.6% and 61.5%, respectively, were employed. Median (interquartile range [IQR]) screen time per week among men and women was 832.0 (368.8–1263.1) and 852.6 (426.0–1307.5) minutes, respectively. Significant differences were observed between men and women in BMI, weight gain, screen time, education level, and employment status. Significant differences were observed between urban and suburban areas in BMI, living arrangements, education level, and household income level; however, no differences in age, marital status, employment status, weight gain, or screen time were observed between areas.

**Table 1.** Sociodemographic characteristics of respondents

	Men		Women			Nerima		Kanuma		
				
	*n*	%	*n*	%	*P* value	*n*	%	*n*	%	*P* value
Overall	540	100.0	494	100.0		548	52.7	491	47.3	
Age group (years)										
40–49	149	27.6	149	30.2	0.645	153	27.9	146	29.7	0.115
50–59	189	35.0	164	33.2		175	31.9	178	36.3	
60–69	202	37.4	181	36.6		220	40.1	167	34.0	
Mean ± SD	55.6 ± 8.4		55.3 ± 8.4		0.637	55.8 ± 8.5		55.1 ± 8.3		0.190
Marital status										
Unmarried	85	15.8	74	15.0	0.731	89	16.3	71	14.5	0.440
Married	453	84.2	419	85.0		458	83.7	418	85.5	
Living condition										
Living with others	493	91.3	465	94.1	0.095	498	90.9	464	94.5	0.032
Living alone	47	8.7	29	5.9		50	9.1	27	5.5	
Education level										
4 or more years of university	246	45.6	90	18.2	<0.001^a^	250	45.6	87	17.7	<0.001^a^
2 years of university or equivalent	51	9.4	160	32.4		120	21.9	93	18.9	
High school or junior high school	243	45.0	244	49.4		178	32.5	311	63.3	
Employment status										
Employed	462	85.6	304	61.5	<0.001^a^	403	73.5	367	74.7	0.671
Not employed	78	14.4	190	38.5		145	26.5	124	25.3	
Household income level (yen)										
<3 000 000	104	19.3	127	25.7	0.066	105	19.2	129	26.3	<0.001^a^
<5 000 000	149	27.6	140	28.3		145	26.5	144	29.3	
<7 000 000	109	20.2	97	19.6		102	18.6	104	21.2	
<10 000 000	104	19.3	78	15.8		108	19.7	76	15.5	
≥10 000 000	74	13.7	52	10.5		88	16.1	38	7.7	
BMI										
<25 kg/m^2^	373	69.1	414	83.8	<0.001^a^	433	79.0	356	72.5	0.016
≥25 kg/m^2^	167	30.9	80	16.2		115	21.0	135	27.5	
Mean ± SD	23.7 ± 3.0		22.3 ± 3.3		<0.001^b^	22.6 ± 3.2		23.4 ± 3.2		<0.001^b^
Weight gain										
<10 kg	376	69.6	422	85.4	<0.001^a^	425	77.7	373	76.6	0.711
≥10 kg	164	30.4	72	14.6		122	22.3	114	23.4	
Mean ± SD	7.1 ± 7.8		3.9 ± 7.5		<0.001^b^	5.3 ± 7.7		5.8 ± 8.0		0.307
Screen time, min/week										
Median (interquartile range)	832.0 (368.8–1263.1)		852.6 (426.0–1307.5)		0.041^c^	840.0 (408.4–1269.8)		848.5 (393.9–1269.2)		0.517^c^

All values are *n* (%) unless indicated otherwise. Groups compared by using the (a) χ^2^ test, (b) *t*-test, or (c) Mann-Whitney U test.

BMI: body mass index.

### Association between screen time and weight status

Median (IQR) screen time/week was 843.8 (403.8–1265.6) among participants with a BMI of less than 25 kg/m^2^ and 842.9 (405.0–1403.3) among those with a BMI of 25 kg/m^2^ or higher. The association between screen time and BMI was not statistically significant (*P* = 0.24). Median screen time per week was greater among participants with a weight gain of 10 kg or more (median = 844.8; IQR, 425.6–1357.5) than among those with a weight gain of less than 10 kg since age 20 (841.8, 386.3–1266.6) (*P* = 0.08).

### Association between screen time and demographic variables

Table 2 shows the results of adjusted logistic regression analysis. Unmarried participants (odds ratio [OR], 2.02; 95% CI, 1.32–3.10) and unemployed participants (OR, 1.63; 95% CI, 1.19–2.23) were significantly more likely to spend more time on screen-based sedentary behavior. Participants aged 40 to 49 years (OR, 0.64; 95% CI, 0.46–0.89) reported less screen time during leisure hours than did those aged 60 to 69 years. Screen time was not significantly associated with sex, living arrangements, education level, or household income level.

**Table 2.** Multiple logistic regression analyses of sociodemographic correlates of screen-based sedentary behavior

	OR	95% CI	*P* value
Sex			
Men	0.99	0.75–1.31	0.96
Women	1.00		
Age group (years)			
40–49	0.64	0.46–0.89	0.01
50–59	0.79	0.57–1.09	0.15
60–69	1.00		
Marital status			
Unmarried	2.02	1.32–3.10	0.001
Married	1.00		
Living condition			
Living with others	1.59	0.89–2.81	0.12
Living alone	1.00		
Educational level			
4 or more years of university	0.80	0.59–1.09	0.16
2 years of university or equivalent	0.97	0.69–1.37	0.86
High school or junior high school	1.00		
Employment status			
Not employed	1.63	1.19–2.23	0.001
Employed	1.00		
Household income level (yen)			
<3 000 000	1.21	0.74–2.00	0.44
<5 000 000	1.15	0.73–1.82	0.55
<7 000 000	1.31	0.82–2.11	0.26
<10 000 000	1.41	0.88–2.26	0.16
≥10 000 000	1.00		

OR: odds ratio.

Odds ratios were calculated after adjustment for all variables listed in the table.

## DISCUSSION

The present study examined the association of sociodemographic and anthropometric factors such as body weight and weight gain with screen-based sedentary behavior among Japanese adults. The results revealed that the level of screen-based sedentary behavior varied between population subgroups: individuals who were older, unmarried, or unemployed reported more screen time during their leisure hours.

The present study focused only on leisure screen time rather than total screen-related sedentary behavior. Sedentary behavior tends to occur at specific time points and in specific contexts. Thus, it is difficult to ascertain all the details of such behavior. Furthermore, sedentary behavior is relatively independent of physical activity, and both sedentary and active behaviors can occur at any time of the day.^5^ In addition, determinants associated with sedentary behavior differ by specific domain, such as leisure time versus working time.^19^ It is thus important to determine when sedentary behavior occurs and to study certain specific time periods, such as leisure time.^20^^,^^21^

The median duration of screen time in the present study was 841.8 minutes/week (about 120.6 minutes/day). In contrast, duration of screen time was 188.0 minutes/day in Scotland^12^ and 235.1 minutes/day in Australia^13^; thus, duration of screen time was shorter in Japan than in other countries. However, the present values for screen time were based on participant recall of the number of days spent on screen time during the previous 7 days and the average amount of screen time on each of those days. Thus, we cannot directly compare the present results with those of studies that used different methods to measure duration of screen time, and our results should be interpreted with caution.

From the present results, association between screen time and weight gain since age 20 were found among Japanese adults. By contrast, BMI was not associated with screen time, because only a small percentage of participants had a BMI of 25 kg/m^2^ or higher. In Japan, although the rate of overweight/obesity has rapidly increased during the past decade,^22^ it is low in comparison with other developed countries. In other developed countries and Japan, body weight is positively associated with the risks of lifestyle-related disease and mortality.^23^ Therefore, it was important to determine the sociodemographic correlates of screen time associated with weight status in the present study.

The present study found a positive association between screen time and age. Because previous studies assessed different age groups of adults, care should be taken in interpreting the present findings and comparing them with the results of earlier studies. Previous studies also reported that screen-based sedentary behavior differed by age^7^^,^^24^^–^^28^; however, those studies found no consistently significant relationship between age and general screen-based sedentary behavior.^17^ The results of the present study show a strong relationship between age and screen time. Therefore, interventions should aim to decrease leisure-time sedentary behavior among older Japanese adults.

Marriage, or a long-term relationship, significantly contributes to individual quality of life.^29^ The present study found that unmarried participants tended to report more screen time. Social support strongly correlates with physical activity level and enhances well-being. Being married is associated with an increased likelihood of engaging in health-promoting behaviors such as exercise, presumably because marital partners exert influence over each other’s behavior.^21^ However, a previous study found that the association between marital status and screen-based sedentary behavior was weak and that more evidence was needed.^17^ Thus, the results of the present study are important evidence for an association between marital status and screen-based sedentary behavior.

A previous study^17^ provided evidence of a positive relationship between TV viewing (as part of screen-based sedentary behavior) and being unemployed, but associations between other sedentary behaviors and employment status were less clear. In the present study, unemployed individuals were more likely to report greater screen time. Studies that examined correlates of physical activity among Japanese adults found that unemployed adults were more likely than employed persons to engage in moderate-to-vigorous physical activity.^30^^,^^31^ Therefore, when designing interventions to promote the health of unemployed persons, measures to decrease screen-based sedentary behavior may be required, in addition to those aimed at increasing an already high level of physical activity.

Living arrangements, sex, education level, and household income level were not associated with screen time in the present study. Participants were not asked for detailed information about the persons they lived with or about the nature of their relationship. Hence, the explanatory power of this information is limited; participants who answered that they lived with other people may be living with parents, siblings, or children, among other possibilities. A previous study^17^ reported that current evidence of an association between living with a child and engaging in sedentary behavior was scant. However, only a limited number of studies have investigated this issue; further studies are needed to confirm any association between screen time and living arrangements.

The present study did not find a statistically significant sex difference in duration of screen-based sedentary behavior. Some previous studies found no evidence of a sex difference in screen-based sedentary behavior^32^^–^^38^; however, other studies reported that the amount of screen time was greater among men than among women.^7^^,^^27^^,^^39^^,^^40^ Therefore, any approach to discourage screen time during leisure hours may need to target both Japanese women and men.

Socioeconomic status may be a key factor in determining the health status of an individual. A systematic review^17^ of data from other countries summarized associations between education, income, and screen-based sedentary behavior. The authors suggested that the association between income and TV viewing was inconclusive, that there was no association between income and amount of computer use, and that educational level may be inversely associated with TV viewing and positively associated with computer use. However, in the present study, neither education status nor household income level were associated with screen time. Therefore, to decrease sedentary behavior in relation to educational status and household income level, an effective approach might be to focus on screen-based sedentary behavior in the relevant segments of the Japanese adult population.

The present study had limitations. First, the cross-sectional nature of the study limits conclusions regarding the causality of observed relationships of sociodemographic and anthropometric factors with screen time. Second, to estimate screen time, the study relied on self-reported measures that are subject to error, owing to different interpretations of the questions.^41^^,^^42^ In addition, the reliability and validity of the scale have yet to be demonstrated in Japan. Third, the study respondents slightly differed from the general population. To estimate the representativeness of participant responses, the adjusted prevalences of marital and employment statuses, by age bracket, were compared with data from the Japanese Population Census Survey of 2005.^43^ In the 2 surveys, the prevalence of married participants was 84.2% and 63.5%, respectively, among men and 85.0% and 63.8% among women. Regarding employment status, 85.6% and 66.3% of men, and 61.5% and 34.9% of women, respectively, worked full-time or part-time.^44^ Therefore, the basic characteristics of respondents might have been biased, and the findings in such a setting may not be applicable to the general population. However, the characteristics of the present population are sufficiently similar to those of the general population because the present study randomly selected participants from a registry of residential addresses of each city, which allowed an equal number of responses to be obtained from both sexes and from each age group category between age 40 and age 69 years. Fourth, the study included only participants aged 40 to 69 years, so the generalizability of the present study findings to other age groups is unclear and requires further assessment.

Despite these limitations, few studies have examined the present topic among a randomly selected Japanese population. The present findings increase understanding of the determinants of screen time and may help in developing new strategies and interventions to promote public health and well-being in Japan.

### Conclusions

The present study identified sociodemographic and anthropometric factors associated with leisure-time screen-based sedentary behavior among Japanese adults. In the absence of similar studies in Japan, the present results will help in developing interventions to promote health and discourage screen-based sedentary behavior by targeting important determinants of sedentary pursuits in all sociodemographic groups, and especially among elderly, unmarried, and unemployed adults.

## ONLINE ONLY MATERIALS

Abstract in Japanese.
