# Supplementary material for: Sociodemographic and Anthropometric Factors Associated With Screen-Based Sedentary Behavior Among Japanese Adults: A Population-Based Cross-Sectional Study
Source: J Epidemiol. 2013 Sep 5;23(5):382–8. doi: 10.2188/jea.JE20130008 (PMC3775533; doi:10.2188/jea.JE20130008)
Supplement: Abstract in Japanese. [file je-23-382-s001.pdf]

日本人成人におけるスクリーンタイムの座位行動に関連する社会人口統計学的要因および身体的特徴：地域住民を対象とした横断研究

石井　香織、柴田　愛、岡　浩一朗
早稲田大学スポーツ科学学術院

背景
健康リスクに影響を与えるスクリーンタイム(テレビやコンピューターの使用)の座位行動に関連する要因の検討が求められている。そこで本研究では、日本人成人におけるスクリーンタイムの座位行動と体重・体格および社会人口統計学的要因との関連を検討した。

方法
日本の2つの地域に居住する40〜69歳の地域住民1,034名に対し横断調査を実施した。自己記入式質問紙にて社会人口統計学的要因、身長、体重およびスクリーンタイムの座位行動時間を調査した。BMIおよび20歳時からの体重増加によるスクリーンタイムの違いをマン・ホイットニーのU検定にて検討した。また、ロジスティック回帰分析により、スクリーンタイムに関連する社会人口統計学的要因を検討した。

結果
平均年齢(標準偏差)は男性で55.6(8.4)歳、女性で55.3(8.4)歳、週あたりのスクリーンタイムの中央値(25パーセンタイル-75パーセンタイル)は男性で832.0(368.8-1263.1)分、女性で852.6(426.0-1307.5)分であった。20歳からの体重増加が10kg以上の者は、10kg未満の者よりスクリーンタイムが長かった(P= 0.08)。独身者、仕事に就いていない者は、よりスクリーンタイムが長かった。また、40〜49歳の者はそれ以上の者よりもスクリーンタイムが短かった。

結論
本研究の結果より、日本人成人におけるスクリーンタイムの座位行動に関連する体重・体格および社会人口統計学的要因が特定された。本研究の結果は、特に高齢者、独身者、仕事に就いていない者に対するスクリーンタイムの座位行動を減少させるための対策の必要性を示している。

キーワード：体重・体格、日本人、座位行動、社会人口統計学
